# Intolerance of uncertainty predicts fear extinction in amygdala-ventromedial prefrontal cortical circuitry

**DOI:** 10.1186/s13587-015-0019-8

**Published:** 2015-07-10

**Authors:** Jayne Morriss, Anastasia Christakou, Carien M. van Reekum

**Affiliations:** Centre for Integrative Neuroscience and Neurodynamics, School of Psychology and Clinical Language Sciences, University of Reading, Earley Gate, Whiteknights Campus, RG6 6AH Reading, UK

**Keywords:** Intolerance of uncertainty, Fear extinction, Amygdala, Prefrontal, fMRI

## Abstract

**Background:**

Coordination of activity between the amygdala and ventromedial prefrontal cortex (vmPFC) is important for fear-extinction learning. Aberrant recruitment of this circuitry is associated with anxiety disorders. Here, we sought to determine if individual differences in future threat uncertainty sensitivity, a potential risk factor for anxiety disorders, underly compromised recruitment of fear extinction circuitry.

Twenty-two healthy subjects completed a cued fear conditioning task with acquisition and extinction phases. During the task, pupil dilation, skin conductance response, and functional magnetic resonance imaging were acquired. We assessed the temporality of fear extinction learning by splitting the extinction phase into early and late extinction. Threat uncertainty sensitivity was measured using self-reported intolerance of uncertainty (IU).

**Results:**

During early extinction learning, we found low IU scores to be associated with larger skin conductance responses and right amygdala activity to learned threat vs. safety cues, whereas high IU scores were associated with no skin conductance discrimination and greater activity within the right amygdala to previously learned safety cues. In late extinction learning, low IU scores were associated with successful inhibition of previously learned threat, reflected in comparable skin conductance response and right amgydala activity to learned threat vs. safety cues, whilst high IU scores were associated with continued fear expression to learned threat, indexed by larger skin conductance and amygdala activity to threat vs. safety cues. In addition, high IU scores were associated with greater vmPFC activity to threat vs. safety cues in late extinction. Similar patterns of IU and extinction learning were found for pupil dilation. The results were specific for IU and did not generalize to self-reported trait anxiety.

**Conclusions:**

Overall, the neural and psychophysiological patterns observed here suggest high IU individuals to disproportionately generalize threat during times of uncertainty, which subsequently compromises fear extinction learning. More broadly, these findings highlight the potential of intolerance of uncertainty-based mechanisms to help understand pathological fear in anxiety disorders and inform potential treatment targets.

## Background

The modulation of affective responses to cues based on their current contextual relevance is crucial for preserving health and protecting against psychopathology [1–3]. Past animal and human research using classical fear conditioning paradigms has demonstrated an important role of the amygdala in fear acquisition and expression, and of the ventromedial prefrontal cortex (vmPFC) in fear extinction [4–6].

During fear acquisition, heightened amygdala activity and increased skin conductance have been observed in response to previously neutral cues that, through conditioning, come to be associated with aversive outcomes (conditioned stimulus, CS+, e.g. shock or tone) [4, 7, 8]. Subsequent extinction training, which involves repeated presentations of the CS+ without the aversive outcome, results in reduced amygdala and skin conductance responsivity over time [5, 9, 7]. The vmPFC is critical for the fear extinction process and the observed reduction in amygdala and skin conductance responses to the CS+ over time [3]. For example, stimulation of the infralimbic cortex in rats, an area homologous to the human vmPFC, reduces responsiveness of amygdala neurons and defensive freezing behavior to conditioned tones [10]. In both humans and animals, increased vmPFC activity to the CS+ has been observed in late extinction phases [6, 11], and during subsequent extinction sessions, conducted a few days after initial fear acquisition [12, 13].

Current exposure therapies for anxiety disorders are based on fear extinction models. A large body of clinical and neurobiological research using fear extinction paradigms has shown that individuals with anxiety/trauma disorders are prone to delayed fear extinction learning or even resistance to fear extinction (for reviews see, [3, 14, 15]). For example, compared to healthy controls, anxiety patients show elevated autonomic nervous system and amygdala responding and reduced recruitment of the vmPFC to both threat and safety cues at the start of extinction and to threat cues across fear extinction learning [16, 11, 17, 18].

In addition to clinical samples, it is important to test fear extinction learning in non-clinically anxious individuals to appropriately separate those processes that are risk factors for anxiety disorder development from those that are consequential to an anxiety disorder. A series of recent studies have shown that individuals with high trait anxiety and genetic predisposition for anxiety exhibit the following: (1) exaggerated autonomic nervous system responding to both threat and safety cues in the early phase of extinction learning [9] and (2) sustained autonomic nervous system responding, sustained amygdala activation and atypical activation in the medial prefrontal cortex to threat cues from the early to late phase of fear extinction learning [19–21, 9]. Genetic evidence also points to similar temporal patterns of delayed fear extinction learning and increased risk for anxiety in both homozygote and heterozygote Met allele carriers of the brain-derived neurotrophic factor (BDNF) Val66Met genotype in mice [21–23] and humans [24, 21, 25]. Furthermore, both the phenotypic and genetic results in mice and humans appear to be specific to fear extinction learning rather than fear acquisition [19, 26, 20, 27, 21–24, 28], but see [27, 9], suggesting that individuals prone to developing an anxiety disorder have difficulty inhibiting learned threat cues and have a tendency to generalize threat to safety cues, rather than being more readily or strongly conditioned [26, 29].

Simple changes to the contingency at the start of fear extinction learning are inherently uncertain and ambiguous. Despite this, the majority of fear extinction studies have focused predominantly on self-reported trait anxiety [20, 19, 9] rather than self-reported intolerance of uncertainty (IU) [30]), a key transdiagnostic factor in maintaining and mediating anxiety and depression [31–34]. IU is defined as a difficulty in accepting the possibility of future negative events, rendering ambiguous or even neutral cues as threatening. In the context of fear extinction learning, changes to contingency may exacerbate future threat uncertainty, resulting in threat responses to both learned threat and safety cues at the start of extinction, and continued threat responses to learned threat cues in late extinction for those individuals who find uncertainty anxiety-provoking. Given the extant literature, it seems pertinent to examine whether IU carries the association between trait anxiety and delayed fear extinction learning. Understanding associations between IU and fear extinction learning could help characterize IU-based maintenance of anxiety, with implications for targeted treatment [35, 34, 30].

Here, we used cued fear conditioning with acquisition and extinction phases to assess the relationship between individual differences in self-reported IU and in psychophysiological and neural correlates of fear extinction learning over time. We measured event-related fMRI, skin conductance response (SCR), pupil dilation and behavioral ratings whilst participants performed the conditioning task. We used an aversive sound as an unconditioned stimulus and visual shapes as conditioned stimuli, as in previous conditioning research [36, 13, 37, 19, 38, 4]. We hypothesized that, during extinction learning, threat uncertainty sensitivity would predict generalized fear expression to both learned threat and safety cues, and/or sustained fear expression to learned threat cues. Given that fear extinction paradigms are temporally sensitive [5, 13, 3, 21, 9, 20], we expected this effect to be indexed by the following: (1) larger responses in high IU individuals to both learned threat and safety cues in *early* fear extinction, across our physiological and behavioral measurements, including relatively higher amygdala activation; (2) sustained larger responses across measures in high IU individuals to learned threat cues vs. safety cues during *late* fear extinction. We further predicted (3) an association between vmPFC activation and the management of responses to threat vs. safety cues during extinction in low IU individuals. We tested the specificity of the involvement of IU by comparing it with broader measures of anxiety, such as Spielberger State-Trait Anxiety Inventory, Trait Version (STAIX-2) [39] and Penn State Worry Questionnaire (PSWQ) [40].

## Methods

### Participants

Twenty-two right-handed volunteers were recruited from the University of Reading and local area through advertizements (M age = 23.59, SD age = 2.75; 12 females and 10 males). All participants had normal or corrected to normal vision and were medication-free. Participants provided written informed consent and received a picture of their brain and £20 for their participation. The University of Reading’s Research Ethics Committee approved the study protocol.

### Conditioning task

Visual stimuli were presented through MRI-compatible VisualSystem head-coil mounted eye goggles (NordicNeuroLab, Bergen, Norway), which displayed stimuli at 60 Hz on an 800 × 600 pixel screen. Sound stimuli were presented through MRI-compatible AudioSystem headphones (NordicNeuroLab, Bergen, Norway). Participants used an MRI-compatible response box with their dominant right hand to respond.

Visual stimuli were blue and yellow squares with 183 × 183 pixel dimensions, resulting in a visual angle of 5.78° × 9.73°. The aversive sound stimulus consisted of a fear inducing female scream (sound number 277) from the International Affective Digitized Sound battery (IADS-2) and which has been normatively rated as unpleasant (M = 1.63, SD = 1.13) and arousing (M = 7.79, SD = 1.13) [41]. We used Audacity 2.0.3 software (http://audacity.sourceforge.net/) to shorten the female scream to 1000 ms in length and to amplify the sound by 15 dB, resulting in a 90-dB (±5 dB) sound.

The three learning phases were presented in three separate blocks. During the acquisition phase, one of two squares (i.e. blue or yellow, counterbalanced) was always paired with the aversive sound (CS+), whilst the other square was presented alone (CS−). In a subsequent extinction phase, both stimuli were presented unpaired (CS+, CS−). A third phase comprised partial reacquisition, where the CS+ square was paired with the sound 25 % of the time and the CS− remained unpaired (not reported here).

Participants were instructed to attend and listen to the stimulus presentations and provide a rating of the stimulus following each trial. The rating scale asked how ‘uneasy’ the participant felt after each stimulus presentation, where the scale ranged from 1 (‘not at all’) to 10 (‘extremely’).

**Fig. 1:**
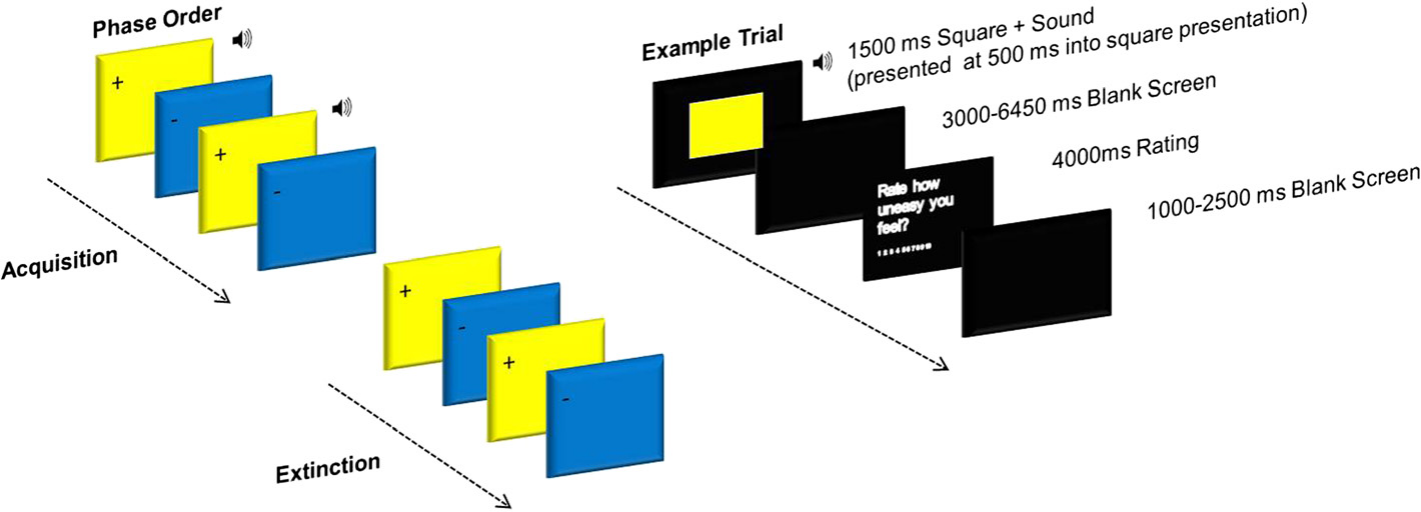
Conditioning task design

The acquisition phase consisted of 24 trials (12 CS+, 12 CS−), the extinction phase 32 trials (16 CS+, 16 CS−) and the reacquisition phase 60 trials (8 CS+, 24 CS+_unpaired_, 28 CS−; data not presented here) (see Fig. 1). Experimental trials were pseudo randomized into an order, which resulted in no more than three presentations of the same stimulus in a row. Colour-sound contingencies were counterbalanced across the sample.

### Procedure

Participants arrived at the laboratory and were informed of the experimental procedures. First, participants completed a consent form as an agreement to take part in the study. Second, a hearing test was performed with an audiometer to check for normative hearing (e.g. 500–8000 Hz, below 30 dB). Third, participants completed a battery of cognitive tasks (results not reported here) and questionnaires on a computer outside of the scanner. Next, participants were taken to the MRI unit. We used a conditioning task inside the scanner, whilst concurrently recording ratings, electrodermal activity and pupil dilation. Participants were simply instructed to: (1) maintain attention to the task by looking and listening to the colored squares and sounds presented, (2) respond to the uneasiness scale using the button box and (3) to keep as still as possible. After scanning, participants rated the sound stimulus outside of the scanner.

### Questionnaires

To assess emotional disposition, we presented the following six questionnaires on a computer: two versions of the Positive and Negative Affect Scales (PANAS-NOW; PANAS-GEN) [42], Spielberger State-Trait Anxiety Inventory, Trait Version (STAIX-2) [39], PSWQ [40], IU [43] and the Barratt Impulsiveness Scale (BIS-11) [44]. We focused on IU because of the intrinsic uncertainty within conditioning paradigms. Similar distributions and internal reliability of scores were found for the anxiety measures, IU (M = 53.04; SD = 15.68; range 27–85; α = .90), STAIX-2 (M = 40.33; SD = 7.92; range = 27–53; α = .85) and PSWQ (M = 41.47; SD = 11.10; range = 20–65; α = .90). We collected the other questionnaires to check for correlational consistency and specificity across anxiety measures as well as to check for outlying values on IU due to mood or impulsivity.

### Sound stimulus rating

Participants rated the valence and arousal of the sound stimulus using 9-point Likert scales ranging from 1 (valence: negative; arousal: calm) to 9 (valence: positive; arousal: excited).

### Behavioral data scoring and reduction

Rating data from the conditioning task were reduced for each participant by calculating their average responses for each experimental condition. Missing data points were excluded.

### Physiological acquisition and reduction

Electrodermal recordings were obtained using ADInstruments (ADInstruments Ltd., Chalgrove, Oxfordshire) hardware and software. An ML138 Bio Amp connected to an ML870 PowerLab Unit Model 8/30 amplified the EDA signal, which were digitized through a 16-bit A/D converter at 1000 Hz. EDA was measured during the scanning session with MRI-safe MLT117F Ag/AgCl bipolar finger electrodes filled with NaCl electrolyte paste (Mansfield R & D, St Albans, Vermont, USA) that were attached to the distal phalanges of the index and middle fingers of the left hand. A constant voltage of 22 mV/ms at 75 Hz was passed through the electrodes, which were connected to a ML116 GSR Amp. SCR were scored when there was an increase of skin conductance level exceeding 0.03 microSiemens. The amplitude of each response was scored as the difference between the onset and the maximum deflection prior to the signal flattening out or decreasing. SCR onsets had to be within 7 s following each trial to be included. Trials with no discernible SCRs were scored zero. The first trial of each experimental phase was excluded, to reduce contamination of averages from the orienting response. SCR amplitudes were square root transformed to reduce skew. Trials with motion artefacts were discarded from the analysis. SCR magnitudes were calculated from remaining trials by averaging SCR square-root-transformed values for each condition.

Pupil dilation was recorded at a sample rate of 60 Hz through a built-in infrared camera on the head-coil mounted eye goggles (NordicNeuroLab, Bergen, Norway). PD data was averaged for each 1000 ms window following stimulus onset, resulting in five windows of 1000 ms each. These data were baseline corrected by subtracting 1000 ms preceding each stimulus onset from a blank screen. Trials were averaged per condition and time window for each participant.

### Learning assessment

To assess whether participants learned the association between the neutral cue and aversive sound, we calculated conditioned response scores for behavioral ratings, pupil dilation and SCR magnitude in extinction. The conditioned response score was the first 2 CS+ trials and the first 2 CS− trials. A positive score indicated a larger response for CS+ vs. CS−, indexing successful conditioning. This type of learning assessment procedure is commonly reported in the fear extinction literature [30, 11, 6, 13]. To reduce subject attrition, we labelled subjects as learners if they had a positive conditioned response score for any measure. Based on the learning assessment criterion, we identified four potential non-learners out of the 22 participants. Since removing the data of these four subjects did not change the results reported here,^1^ we retained the data of all participants.

### Ratings and psychophysiology analysis

IU differences across extinction were assessed by conducting a condition (CS+, CS−) × time (early, late) × IU repeated measures ANCOVA for behavioral ratings, SCR magnitude and pupil dilation. IU was entered as a continuous mean centered predictor variable. The early part of extinction was defined as the first eight CS+ and eight CS− trials, and the last part of extinction was defined as the last eight CS+ and eight CS− trials. For pupil dilation, which was based on second-by-second averaging, we also included the factor window with five levels representing seconds post-stimulus onset. To check for specificity of findings with IU in extinction, we conducted a condition (CS+, CS−) × window × IU repeated measures ANCOVA on behavioral ratings, SCR magnitude and pupil dilation obtained in the acquisition phase.

We performed hierarchical regression analyses on the resulting significant SCR magnitude and pupil dilation difference scores (CS+ − CS− early; CS+ − CS− late; CS+ early − CS+ late; CS− early − CS− late) for extinction and the anxiety measures to test for IU-specific effects. We entered STAIX-2 and PSWQ in the first step and then IU in the second step.

### MRI

Participants were scanned with a 3T Siemens Trio set up with a 12-channel head coil (Siemens Inc., Erlangen, Germany). Three T2*-weighted echo planar imaging (EPI) functional scans were acquired for each phase of the conditioning task consisting of 161, 208, and 380 volumes, respectively (TR = 2000 ms, TE = 30 ms, flip angle = 90°, FOV = 192 × 192 mm, 3 × 3 mm voxels, slice thickness 3 mm with an interslice gap of 1 mm, 30 axial slices, interleaved acquisition).

Following completion of the functional scans, fieldmap and structural scans were acquired, which comprised of a high-resolution T1-weighted anatomical scan (MP-RAGE, TR = 2020 ms, TE = 2.52 ms, flip angle = 90°, FOV = 256 × 256 mm, 1 × 1 × 1 mm voxels, slice thickness 1 mm, sagittal slices), two fieldmaps (TR = 488 ms, TE 1 = 4.98 ms, TE 2 = 7.38 ms, flip angle = 60°, FOV = 256 × 256 mm, slice thickness 4 mm with an interslice gap of 4 mm, 30 axial slices) and diffusion weighted images, which will not be further discussed here (TR = 6800 ms, TE = 93 ms, flip angle = 60°, FOV = 192 × 192 mm, slice thickness 2 mm with an interslice gap of 2 mm, *b*-value = 1000, 64 axial slices, 30 diffusion gradients).

### fMRI analysis

FMRI analyses were carried out in Feat version 5.98 as part of FSL (FMRIB’s Software Library, www.fmrib.ox.ac.uk/fsl). Brains were extracted from their respective T1 images by using the FSL brain extraction tool (BET) [45]. Distortion, slice timing and motion correction were applied to all extracted EPI volumes using FUGUE and MCFLIRT tools. Gaussian smoothing (FWHM 5 mm) and a 50 s high pass temporal filter were applied.

A first-level GLM analysis was carried out for each functional scan run from acquisition and extinction. Separate regressors were specified for the experimental conditions of primary interest in each learning phase (acquisition: CS+>CS−, extinction: CS+>CS−) by convolving a binary boxcar function with an ideal haemodynamic response (HR), which corresponded to the length of each trial (1500 ms). Regressors for the uneasiness rating period and six motion parameters were included to model out brain activity that was unrelated to the conditions of interest.

We defined two main effect contrasts to reveal fear extinction-related activity. To examine temporal effects across extinction, we contrasted (CS+ vs. CS−)_early_ > (CS+ vs. CS−)_late_. We defined early extinction as the first eight trials for CS+ and CS− and the last eight trials for CS+ and CS−. Particular focus is given to the temporal effects across extinction, given our predictions. We also examined the overall effect of CS+ vs. CS− during extinction for comparison against the extant literature. All contrasts were normalized and registered to MNI standard space using FLIRT [46]. Second-level GLM analysis consisted of regressors for the group mean and demeaned IU scores using FSL’s FLAME stage 1 + 2 procedure. Whole-brain analysis was carried out using cluster thresholding with a *z* = 2.3 and a corrected *p* < 0.05.

We were specifically interested in the extent to which IU scores would be associated with the BOLD response in the amygdala and vmPFC for early and late extinction phases. Therefore, we performed small volume corrections on the left amygdala, right amygdala and vmPFC using cluster thresholding with a *z* = 2.3 and a corrected *p* < 0.05 on the IU × (CS+ vs. CS−)_early_ > (CS+ vs. CS−)_late_ extinction contrast map. We used anatomically defined masks from the Harvard-Oxford cortical and subcortical structural atlases in FSL [47]. We selected the left amygdala, right amygdala and frontal medial cortex regions with a 50 % probability threshold. For control purposes, we also applied small volume corrections within the left amygdala, right amygdala and vmPFC on the IU × acquisition CS+ vs. CS− and the IU × extinction CS+ vs. CS− contrast maps.

To assess fear expression correspondence between the amygdala and psychophysiology measures, we correlated percent BOLD signal response from significant amygdala regions and SCR magnitude/pupil dilation.

We performed hierarchical regression analyses on the resulting statistical a priori regions of interest difference scores from extinction (CS+ − CS− early; CS+ − CS− late; CS+ early − CS+ late; CS− early − CS− late) and the anxiety measures to test for IU-specific effects, STAIX-2 and PSWQ in the first and then IU in the second step.

## Results

One participant’s data were removed from all analyses due to having an extreme IU score that was +3 SD from the group mean.

### Questionnaires

As expected, the anxiety measures were positively correlated with each other, suggesting shared variance, IU with PSWQ, *r*(19) = .590, *p* = .005, IU with STAIX-2, *r*(19) = .619, *p* = .003, and PSWQ with STAIX-2, *r*(19) = .657, *p* = .001.

### Ratings

Participants rated the sound stimulus serving as the US as negative (*M* = 3.52, *SD* = 1.63) and moderately arousing (*M* = 5.23, *SD* = 2.14). With respect to the uneasiness ratings (on a scale from 1 to 10), a main effect of condition was found for acquisition across all individuals, *F*(1,19) = 13.394, *p* = .002. During acquisition, participants significantly reported feeling more uneasy for the CS+ relative to the CS− trials, *p* = .002 (for descriptive statistics, see Table 1). We found no effect of condition or condition × time for the uneasiness ratings during extinction, *p*’s > .1, *F*’s < 1 (see Table 1). Results revealed no IU differences for uneasiness ratings for any of the experimental phases, *p*’s > .3, *F*’s > .1, max *F* = 1.015.

**Table 1:** Summary of means (SD) for each dependent measure as a function of condition and phase

	Acquisition		Extinction		Early extinction		Late extinction	
Measure	CS+	CS−	CS+	CS−	CS+	CS−	CS+	CS−
Physiological								
Square root transformed SCR magnitude (μS)	.27 (.17)**	.13 (.11)**	.16 (.13)*	.13 (.12)*	.20 (.17)	.14 (.11)	.13 (.14)	.11 (.14)
Pupil dilation (Δmm)	−.023 (.010)	−.024 (.010)	−.025 (.008)	−.024 (.013)	−.027 (.015)	−.026 (.018)	−.023 (.008)	−.023 (.022)
Behavioral								
Uneasiness rating (1–9)	3.61 (1.93)**	2.09 (1.50)**	1.67 (1.23)	1.75 (1.32)	1.84 (1.27)	1.88 (1.42)	1.49 (1.38)	1.41 (1.31)

Note: SCR magnitude (μS), skin conductance magnitude is measured in microSiemens. Pupil dilation (Δmm) is measured in delta millimetres. Significant comparisons are specified with *p < .05 and **p < .01

### SCR magnitude

Seven subjects were removed from the SCR magnitude analysis due to six subjects not responding, which is not uncommon when recorded in an MRI setting (see ‘Methods’ section), and one subject with a recording error.

As expected, larger SCR magnitudes were found for CS+ vs. CS− during acquisition, *F*(1,12) = 14.376, *p* = .003 (see Table 1), but there was no interaction between condition × IU, *F*(1,12) = .564, *p* = .467.

**Fig. 2:**
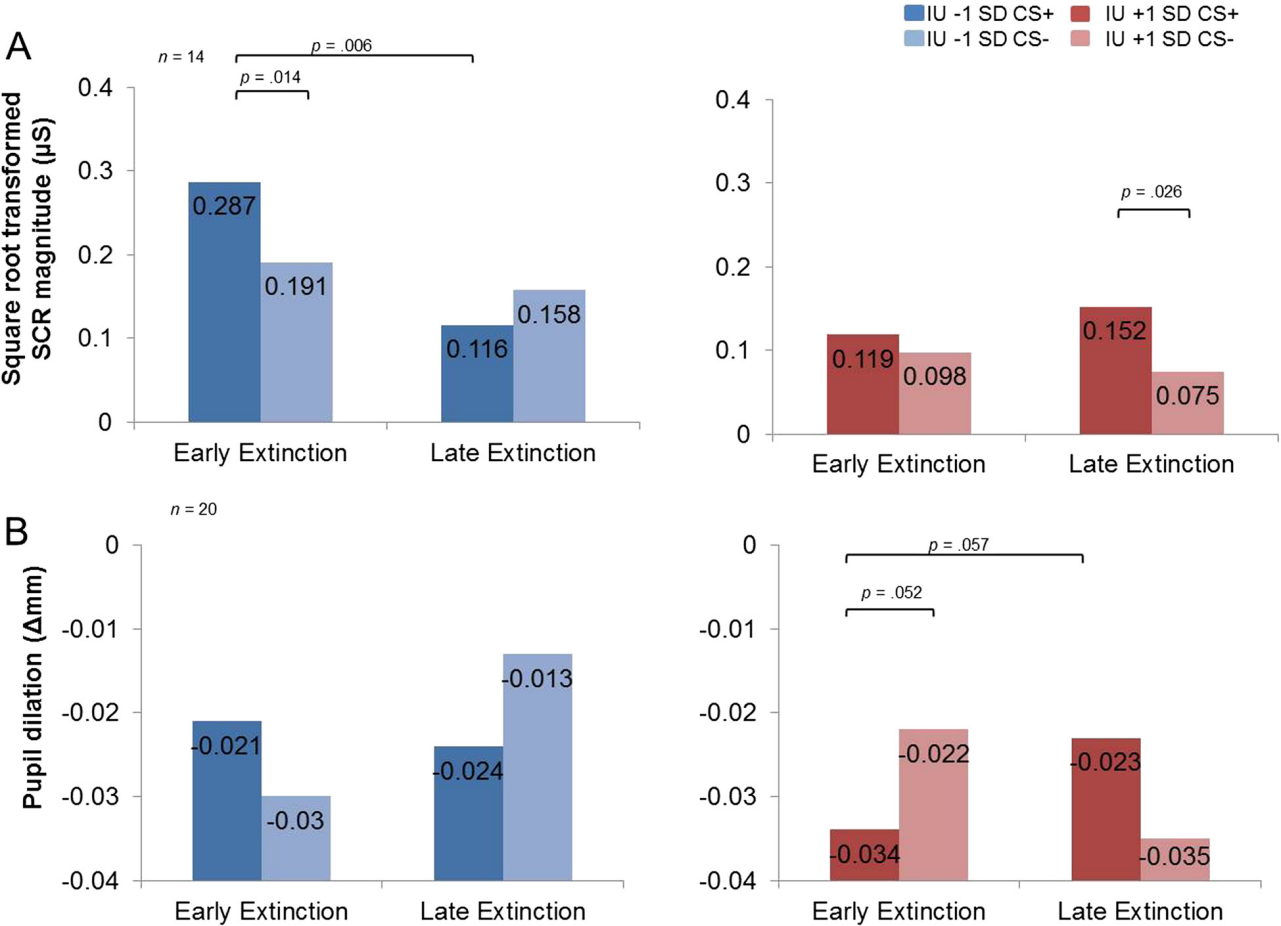
Intolerance of uncertainty predicts psychophysiology during fear extinction. Bar graphs depicting IU differences ±1 SD from the mean during early and late extinction learning. **a** SCR magnitude and **b** pupil dilation. Low IU were associated with significantly greater SCR magnitude responses to CS+ vs. CS− in early extinction and no differences between stimuli in late extinction. High IU scorers showed no differences in SCR magnitude to CS+ and CS− stimuli in early extinction, and delayed discrimination in SCR magnitude to CS+ vs. CS− in late extinction. The pupil dilation results followed a similar pattern to the SCR magnitude results, albeit at trend. SCR magnitude (μS), skin conductance magnitude measured in microSiemens; Pupil dilation (Δmm) measured in delta millimetres

During extinction, we found greater SCR magnitude for the CS+ vs. CS−, *F*(1,12) = 5.369, *p* = .039 (see Table 1), but no significant interaction effect between condition and time, *F*(1,12) = 1.711, *p* = .215. However, as predicted, we found a significant condition × time × IU interaction, *F*(1,12) = 8.782, *p* = .012. Further inspection of follow-up pairwise comparisons for early vs. late extinction at IU ±1 SD from the mean revealed that at the low IU end (1 SD below the IU mean) is associated with the commonly reported extinction pattern, including discrimination between CS+ and CS− in early extinction, *p* = .026, but no significant differences between CS+ and CS− in late extinction, *p* = .139 (see Fig. 2a). Furthermore, low IU is associated with a reduction in SCR magnitude to the CS+ from early to late extinction, *p* = .006, but not to the CS− from early to late extinction, *p* = .425. High IU (captured at 1 SD above the mean) is associated with the opposite pattern, with no significant differences between CS+ and CS− in early extinction, *p* = .586, but discrimination between CS+ and CS− in late extinction, *p* = .014 (see Fig. 2a). In addition, high IU is not associated with differences in SCR magnitude between CS+ from early to late extinction, *p* = .525, and CS− from early to late extinction, *p* = .582. No other significant main effects or interactions were found with IU, max *F* = 3.552, *p*’s > .08.

We conducted hierarchical regression analyses on the effects that were significant in the ANCOVA above, creating difference scores by subtracting response to CS− from CS+. Hierarchical regression analyses of early and late SCR magnitude difference scores in extinction revealed mixed specificity with IU over the STAIX-2 and PSWQ measures: (1) CS+ − CS− early extinction, first step: *R*^2^ = .409, *F*(2,11) = 1.108, *p* = .364, second step: Δ*R*^2^ = .419, *F*(1,10) = .101, *p* = .757, (2) CS+ − CS− late extinction, first step: *R*^2^ = .390, *F*(2,11) = .986, *p* = .404, second step: Δ*R*^2^ = .755, *F*(1,10) = 9.737 *p* = .011, and (3) CS+ early − CS+ late extinction, first step: *R*^2^ = .620, *F*(2,11) = 3.426, *p* = .70, second step: Δ*R*^2^ = .664, *F*(1,10) = 1.023, *p* = .336.

### Pupil dilation

One subject was removed from the pupil dilation analysis due to a recording error, leaving 20 participants. No effect of acquisition or extinction was found for the whole sample, *p*’s > .1, *F*’s < .2, max *F* = 1.615 (see Table 1). We found a significant condition × time × IU interaction for pupil dilation during extinction, *F*(1,18) = 7.921, *p* = .011. Follow-up pairwise comparisons for early vs. late at IU ±1 SD from the mean showed this effect to be driven by high IU scores, which were associated with greater relative pupil constriction for CS− relative to CS+ at trend during early extinction, *p* = .052, but did not display significant differences between CS+ and CS− in late extinction, *p* = .134 (see Fig. 2b). Furthermore, high IU was characterized by an increase in pupil constriction to the CS+ from early to late extinction at trend, *p* = .057, but not to the CS− from early to late extinction, *p* = .167. Low IU scores (1 SD below the mean) were not associated with significant differences between condition and time, *p*’s > .065 (see Fig. 2b). No other significant interactions were found with IU, *p*’s > .1, max *F* = 1.817.

Following up on the significant effects from the ANCOVA above, hierarchical regression analyses of early and late pupil dilation difference scores in extinction revealed specificity for IU over the STAIX-2 and PSWQ measures: (1) CS+ − CS− early extinction, first step: *R*^2^ = .246, *F*(2,17) = .547, *p* = .589, second step: Δ*R*^2^ = .646, *F*(1,16) = 9.772, *p* = .007, (2) CS+ early − CS+ late extinction, first step: *R*^2^ = .075, *F*(2,17) = .048, *p* = .953, second step: Δ*R*^2^ = .476, *F*(1,16) = 4.565, *p* = .048.

### fMRI

Likely because we had a large individual variation in response patterns during extinction, our whole-brain analyses did not yield significant BOLD differences in our a priori brain regions of interest often reported in the extinction literature [4, 5, 13, 6].^2^ We did, however, find greater lateral occipital cortex and parietal lobule activation across extinction for the CS+ > CS− (see Table 2) as well as greater occipital pole activation in early extinction for the CS+ > CS−, relative to late extinction for the CS+ > CS−, suggesting increased attention for the conditioned stimulus.

**Table 2:** Significant activation patterns in a priori regions of interest and other brain regions during extinction

Extinction	Brain region	BA	Voxels (mm^3^)	Max Z	Location of max Z		
					x	y	z
A priori regions							
(CS+ > CS−)_early_ > (CS+ > CS−)_late_ × IU	R amygdala		33	2.96	26	−8	−12
(CS− > CS+)_early_ > (CS− > CS+)_late_ × IU	R L vmPFC	10	40	2.92	−8	42	−16
Outside a priori regions							
CS+ > CS−	L lateral occipital cortex, inferior parietal lobule	7/39	439	3.31	−38	−60	44
(CS+ > CS−)_early_ > (CS+ > CS−)_late_	R occipital pole	18	643	3.88	34	−94	2
(CS− > CS+)_early_ > (CS− > CS+)_late_	R precentral gyrus, postcentral gyrus	3–4/6	504	3.49	38	−24	38
(CS− > CS+)_early_ > (CS− > CS+)_late_ × IU	Cingulate gyrus, juxtapositional lobule, precentral gyrus, postcentral gyrus, parietal lobule	3–7/40	4267	3.99	−2	−8	60
(CS− > CS+)_early_ > (CS− > CS+)_late_ × IU	R central opercular cortex	6	361	3.16	56	−2	6
(CS− > CS+)_early_ > (CS− > CS+)_late_ × IU	L parietal operculum cortex	40	304	3.16	−52	−28	14
(CS− > CS+)_early_ > (CS− > CS+)_late_ × IU	R parietal operculum cortex	40	292	3.33	56	−26	18
(CS− > CS+)_early_ > (CS− > CS+)_late_ × IU	L cerebellum		274	3.29	12	−70	−18
(CS− > CS+)_early_ > (CS− > CS+)_late_ × IU	R lateral occipital cortex	37	259	3.23	46	−60	−8

Note: clusters for small volume corrected a priori regions and whole-brain corrected regions outside a priori regions corrected for multiple comparisons at *p* < 0.05. Location of cluster’s maximum Z are in MNI space. *BA* Brodmann areas, *R* right, *L* left

**Fig. 3:**
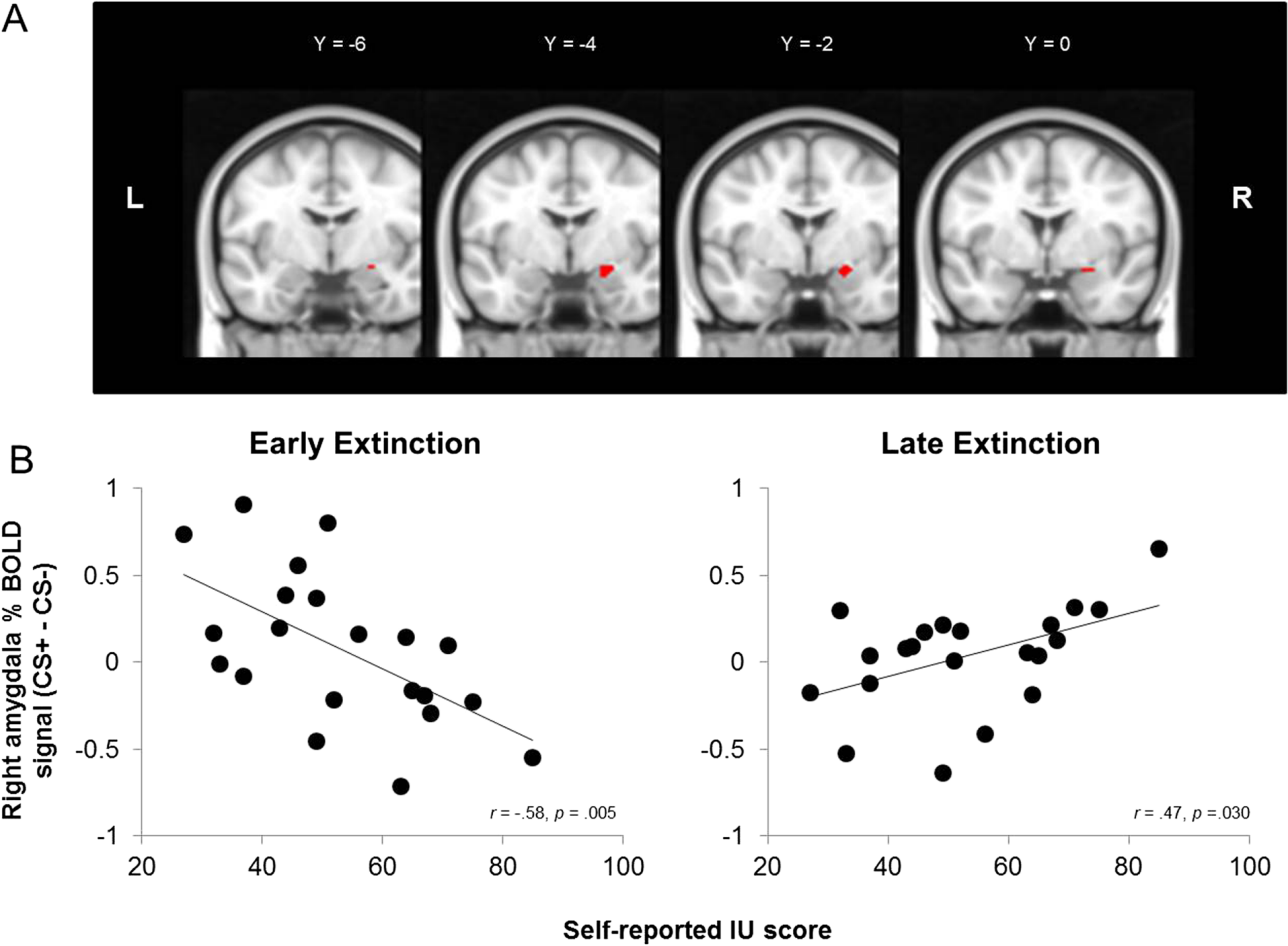
Intolerance of uncertainty predicts right amygdala activation during fear extinction. **a** Right amygdala small volume correction from the (CS− > CS+)_early_ > (CS− > CS+)_late_ × IU contrast in extinction. **b** Significant correlations between percent signal change in the right amygdala for CS+ − CS− and IU scores during early and late extinction. High IU was associated with threat-like responses in the amygdala to CS− in early extinction and to CS+ in late extinction. These findings suggest high IU scorers generalize threat when faced with uncertainty, resulting in compromised safety learning. MNI coordinates: *R* right, *L* left

**Fig. 4:**
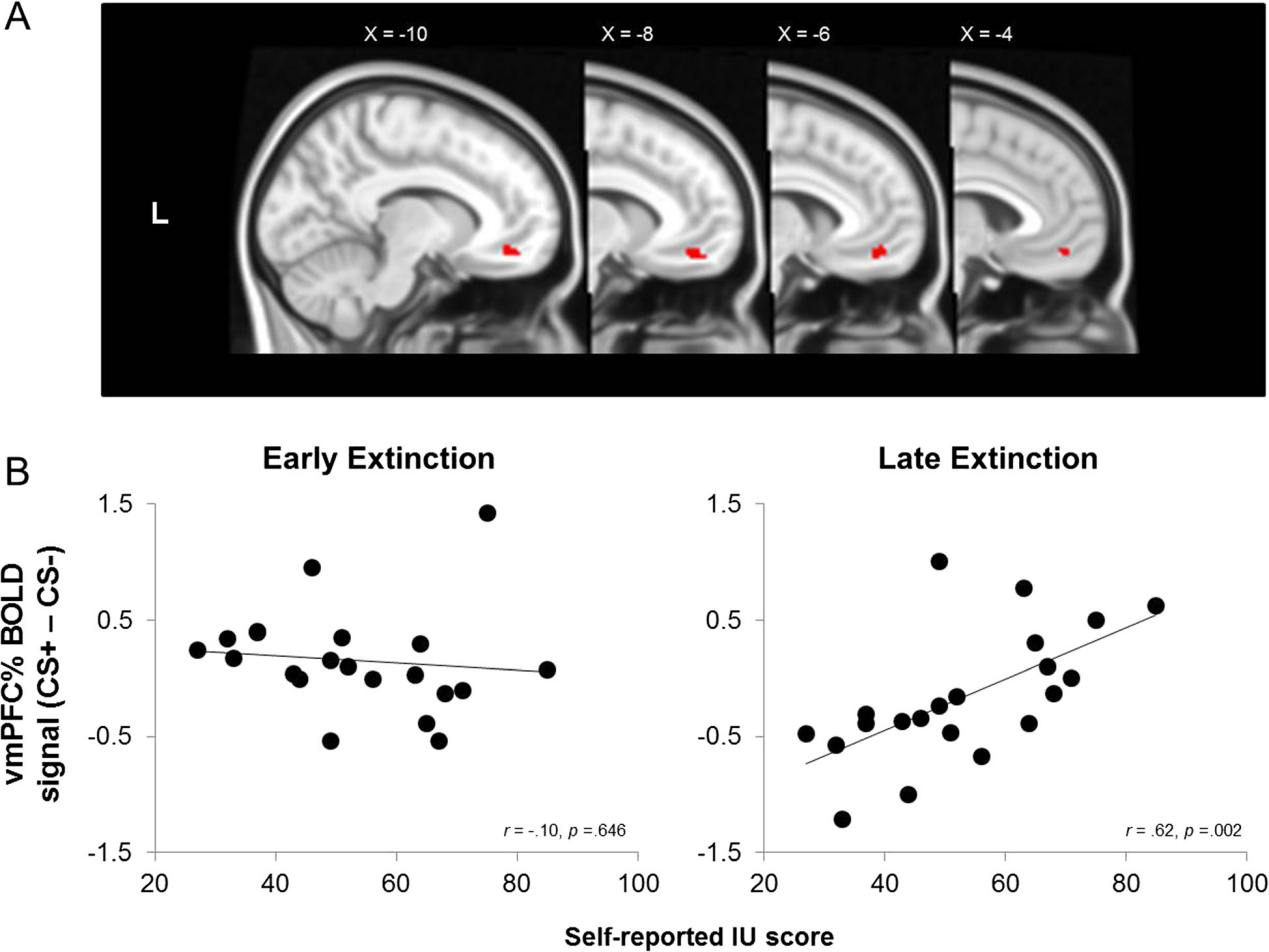
Intolerance of uncertainty predicts vmPFC activation during fear extinction. **a** vmPFC small volume correction from the (CS− > CS+)_early_ > (CS− > CS+)_late_ × IU contrast in extinction. **b** Significant correlations between percent signal change in the vmPFC for CS+ − CS− and IU scores during early and late extinction. During late extinction, high IU scores were associated with increased recruitment of the vmPFC to the CS+, relative to the CS−, suggesting attempts to down regulate fearful associations. MNI coordinates: *R* right, *L* left

As expected, areas within the right amygdala and the vmPFC significantly correlated with IU scores during extinction (see Table 2, Figs. 3 and 4). We performed follow-up correlations to identify the source of the interaction effect from the significant IU × (CS+ vs. CS−)_early_ > (CS+ vs. CS−)_late_ contrast. During early extinction, higher IU predicted increased activation to the CS−, relative to CS+ for the right amygdala cluster, *r*(19) = −.58, *p* = .005 (see Fig. 3). There were no significant effects of IU in the vmPFC cluster during early extinction however, *r*(19) = −0.106, *p* = .646. During late extinction, IU was positively associated with activation to the CS+ relative to the CS− for the right amygdala cluster, *r*(19) = .47, *p* = .030 (see Fig. 3), and, unexpectedly, for the vmPFC cluster, *r*(19) = .62, *p* = .002 (see Fig. 4). In addition, higher IU predicted relative higher right amygdala activity from CS− early to CS− late, *r*(19) = .631, *p* = .002, suggesting generalization of threat to the CS− at the start of extinction. All other condition and time difference scores were not significant for the right amygdala and vmPFC, *p*’s > .125. Furthermore, the BOLD response in areas associated with vigilance, such as the opercular cortex, cingulate gyrus, lateral occipital cortex and precentral gyrus, significantly differed over time as a function of IU scores during extinction (see Table 2).

A hierarchical regression analysis confirmed the significant extinction difference scores from the right amygdala and vmPFC were specific to IU vs. STAIX-2 and PSWQ; adding IU in the second step significantly improved the model: (1) right amygdala for CS+ − CS− early extinction, first step: *R*^2^ = .191, *F*(2,18) = .2.123, *p* = .149, second step: Δ*R*^*2*^ = .404, *F*(1,17) = 6.090, *p* = .025, (2) right amygdala for CS+ − CS− late extinction, first step: *R*^2^ = .099, *F*(2,18) = .987, *p* = .392, second step: Δ*R*^2^ = .237, *F*(1,17) = 3.067, *p* = .098, (3) right amygdala CS− early vs. CS− late extinction, first step: *R*^2^ = .334, *F*(2,18) = 1.127, *p* = .346, second step: Δ*R*^2^ = .642, *F*(1,17) = 8.692, *p* = .009, and (4) vmPFC for CS+ vs. CS− late extinction, first step: *R*^2^ = .122, *F*(2,18) = 1.255, *p* = .309, second step: Δ*R*^2^ = .396, *F*(1,17) = 7.694, *p* = .013.

We found no significant effects of IU during acquisition on a whole-brain basis or within the a priori ROIs. Furthermore, we found no significant effects of IU across the entire extinction phase (early and late collapsed) on a whole-brain basis, nor within the a priori ROIs.

### Relationships between right amygdala and psychophysiology

**Fig. 5:**
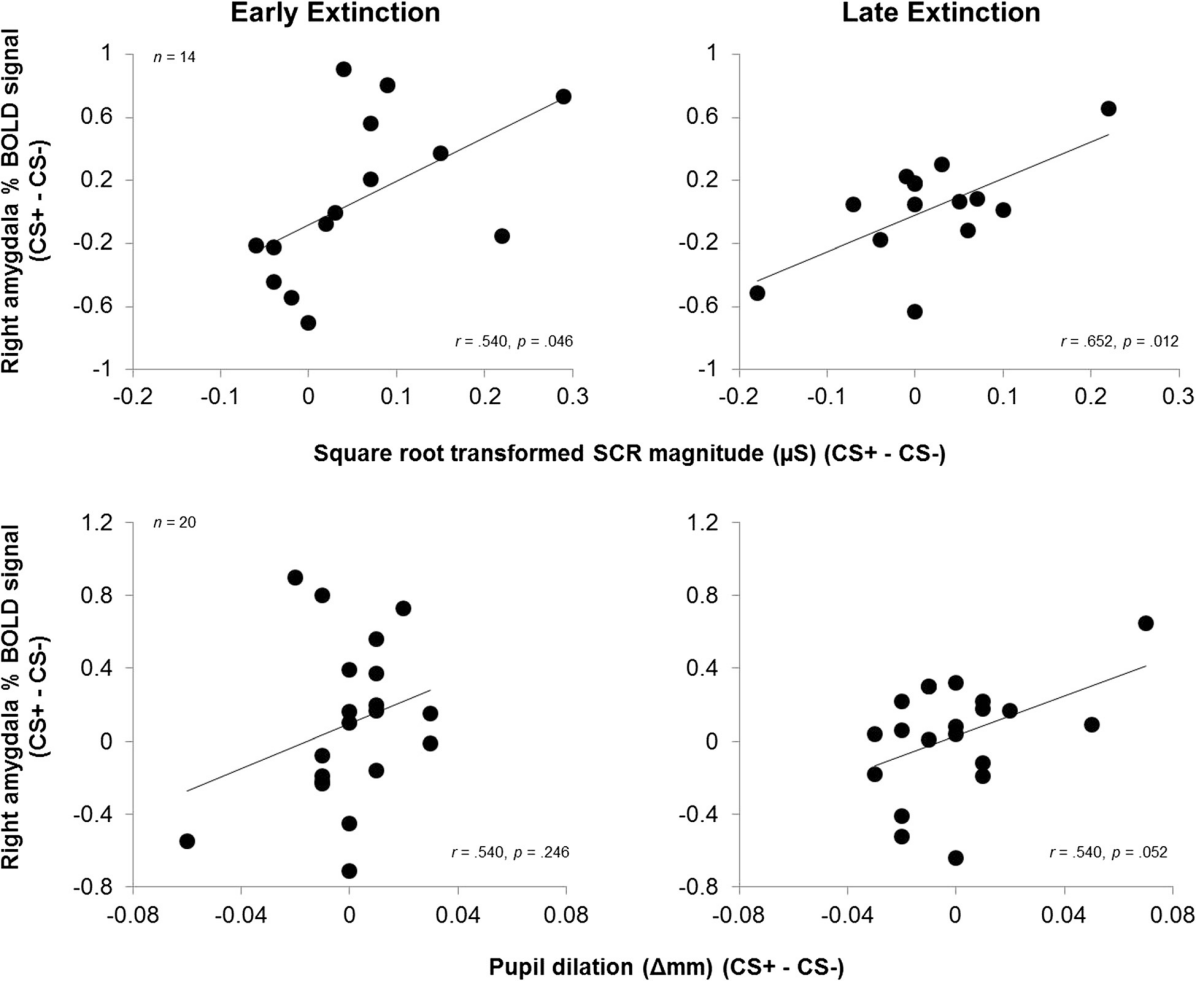
Correlations between percent signal change in the right amygdala and psychophysiology measures. Correlations between percent signal change in the right amygdala and psychophysiology measures. The response in the right amygdala is significantly correlated with SCR magnitude and at trend with pupil dilation, suggesting correspondence between measures of fear expression. SCR magnitude (μS), skin conductance magnitude measured in microSiemens; pupil dilation (Δmm) measured in delta millimetres

Percent BOLD signal difference (CS+ vs. CS−) in the right amygdala correlated positively with SCR magnitude during early, *r*(12) = .540, *p* = .046, and late extinction, *r*(12) = .652, *p* = .012.(see Fig. 5). Percent BOLD signal in the right amygdala was not correlated with pupil dilation during early extinction, *r*(18) = .540, *p* = .246, but did correlate positively during late extinction, *r*(18) = .540, *p* = .052 (see Fig. 5).

### Relationships between a priori ROIs and ratings

Uneasiness rating difference scores for early and late fear extinction did not significantly correlate with percent BOLD signal difference scores for early and late extinction in the a priori ROIs, *p*’s > .35.

## Discussion

We show that self-reported IU, a personality trait implicated in the maintenance of anxiety and depressive disorders [32, 33, 31], predicts psychophysiological and neural recruitment during fear extinction learning. Our data suggest that individuals who are sensitive to threat uncertainty (high IU) are prone to generalize threat, and have difficulty inhibiting learned threat cues, as indexed by heightened psychophysiology and by amygdala and vmPFC function during fear extinction learning. Importantly, our results highlight threat uncertainty sensitivity as a potential factor in the maintenance of extinction-resistant fear, seen in anxiety disorders. Furthermore, these fMRI results were specific to an association between extinction and IU, and did not generalize to other anxiety measures (STAIX-2, PSWQ) or associative learning phases (acquisition).

In early extinction, low IU was characterized by a discrimination of threat and safety cues, consistent with previous fear extinction studies [13, 6, 11] where SCR magnitude and right amygdala response was larger to threat cues, relative to safety cues. Expanding previous research on individual differences in trait anxiety [21, 19, 20, 9, 28, 27] and IU [30], high IU was associated with fear expression to both learned threat and safety cues in early extinction, indexed by indiscriminate SCR magnitude. Furthermore, high IU was associated with larger pupil dilation (at trend) and right amygdala activity to safety vs. threat cues in early extinction. These results suggest potential spill over of learned threat to safety cues in those who are sensitive to future threat uncertainty.

During late extinction, low IU predicted reduced SCR magnitude and right amygdala activity to threat vs. safety cues, suggesting successful fear extinction, in line with previous extinction research [13, 11, 6]. However, high IU predicted larger SCR magnitude, pupil dilation (at trend) and right amygdala to threat vs. safety cues during late extinction, suggesting sustained fear expression to learned threat cues. Although we predicted low IU to be associated with increased vmPFC recruitment to threat vs. safety cues during early extinction, we instead found that high IU was associated with increased vmPFC activation in response to threat vs. safety cues in late extinction. Whilst this pattern was not predicted, it is similar to previous studies that report hyperactivity of the prefrontal cortex during fear extinction for trait anxious individuals [19] and during emotion regulation tasks for depressed patients [48]. Overall, these findings suggest that high IU is associated with slower discrimination of threat from safety cues, which subsequently compromises fear extinction learning.

Notably, we found the fear extinction learning results to be specific to IU, over other broader measures of trait anxiety and worry (STAIX-X2 and PSWQ). The specificity of IU was strongly supported by neural indices and partially supported in SCR magnitude and pupil dilation. Crucially, these results suggest uncertainty to be an important factor in maintaining learned fearful associations and hindering the formation of new safety associations. Furthermore, these data provide initial evidence that uncertainty may be the driver behind previous trait anxiety and fear extinction learning findings [19–21, 9]. These results call for further study of the neural basis underlying uncertainty-based maintenance of anxiety disorders, which may prove useful for clinicians in improving and developing therapies.

We found no evidence of IU predicting differential recruitment of brain regions involved in fear acquisition for the threat and safety cues. However, we used a 100 % reinforcement schedule in the acquisition phase, where the CS+ and US are confounded. Furthermore, the 100 % reinforcement schedule is very certain and unambiguous. Therefore, high IU individuals are not generally more aroused to the US and do not generalize fear to CS− cues during acquisition, at least during 100 % reinforcement. Further work needs to specifically test whether high IU individuals also show discriminatory deficits during the acquisition of conditioned fear [30].

Individual differences in IU were reflected in physiological and brain indices during extinction. However, self-reported arousal ratings did not reflect individual differences in IU in our sample. Divergence between self-reported and neural measures are often reported, perhaps due to lack of direct mapping between behavior and brain activity or to a lack of sensitivity of self-report metrics to capture such individual differences. Interestingly, neural indices during fear extinction were better predicted by IU, over self-reported uneasiness ratings. Such findings suggest IU to be a more suitable predictor of neutral activity during fear extinction than moment-to-moment subjective ratings of uneasiness. However, the lack of relationship between neural activity and subjective ratings may be simply due to the time between phasic cue events and rating periods.

## Conclusions

We found individual differences in IU to specifically predict fear extinction capacity and associated responsivity in psychophysiology and amygdala-vmPFC circuitry. Individuals with high IU scores exhibited exaggerated amygdala and psychophysiology responses to both threat and safety cues during fear extinction. These findings suggest reduced flexibility in amygdala-vmPFC circuitry for high IU individuals. Importantly, these results were specific to IU, highlighting an opportunity for further examination of IU in relation to: (1) current exposure-based therapies, and (2) focused forms of anxiety disorder treatment that target uncertainty-based maintenance of anxiety/fear, such as intolerance of uncertainty therapy [34, 35].

## Abbreviations

Ag/AgCl: silver/silver chloride
ANCOVA: analysis of covariance
BOLD: blood oxygenation level dependent
CS+/CS: conditioned stimulus
EPI: echo planar imaging
FLAME: FMRIB’s local analysis of mixed effects
FLIRT: FMRIB’s linear image registration tool
FMRI: functional magnetic resonance imaging
FMRIB: Oxford Centre for Functional Magnetic Resonance Imaging of the Brain
FOV: field of view
FSL: FMRIB software library
FUGUE: FMRIB’s utility for geometrically unwarping EPIs
GLM: general linear model
IADS-2: international affective digitized sound battery 2
IU: intolerance of uncertainty
M: mean
MCFLIRT: motion correction using FMRIB’s linear image registration tool
MNI: Montreal neurological institute
PSWQ: Penn State Worry Questionnaire
SCR: skin conductance response
SD: standard deviation
STAIX-2: Spielberger State-Trait Anxiety Inventory
TR: repetition time
vmPFC: ventromedial prefrontal cortex

## Footnotes

1 Results do not change when non-learners are removed: the main effect of condition for SCR magnitude during fear extinction learning, without non-learners *F*(1,10) = 7.624, *p* = .020. Condition × time × IU interaction for SCR magnitude during fear extinction learning without non-learners, *F*(1,10) = 8.380, *p* = .016. Extinction CS+ − CS− difference scores for early and late extinction in the right amygdala correlated with IU: early extinction without non-learners, *r*(15) = −.66, *p* = .003. Late extinction without non-learners, *r*(15) = .71, *p* = .001.

2 The CS+ > CS− contrast map revealed vmPFC (approx. 20 voxels) and left amygdala (approx. 4 voxels) clusters at sub-threshold, *z* = 2.0, *p* = .045.

## References

[1] Davidson RJ (1998). Affective style and affective disorders: perspectives from affective neuroscience. Cognition & Emotion.

[2] Frijda NH (1986). The emotions.

[3] Milad MR, Quirk GJ (2012). Fear extinction as a model for translational neuroscience: ten years of progress. Annu Rev Psych.

[4] Büchel C, Morris J, Dolan RJ, Friston KJ (1998). Brain systems mediating aversive conditioning: an event-related fMRI study. Neuron.

[5] LaBar KS, Gatenby JC, Gore JC, LeDoux JE, Phelps EA (1998). Human amygdala activation during conditioned fear acquisition and extinction: a mixed-trial fMRI study. Neuron..

[6] Milad MR, Wright CI, Orr SP, Pitman RK, Quirk GJ, Rauch SL (2007). Recall of fear extinction in humans activates the ventromedial prefrontal cortex and hippocampus in concert. Bio Psych.

[7] Knight DC, Smith CN, Cheng DT, Stein EA, Helmstetter FJ (2004). Amygdala and hippocampal activity during acquisition and extinction of human fear conditioning. Cogn. Affect. Behav. Neurosci..

[8] Neumann DL, Waters AM, Westbury HR (2008). The use of an unpleasant sound as the unconditional stimulus in aversive Pavlovian conditioning experiments that involve children and adolescent participants. Behav. Res. Methods.

[9] Gazendam FJ, Kamphuis JH, Kindt M (2013). Deficient safety learning characterizes high trait anxious individuals. Biol. Psychol..

[10] Milad MR, Quirk GJ (2002). Neurons in medial prefrontal cortex signal memory for fear extinction. Nature.

[11] Milad MR, Pitman RK, Ellis CB, Gold AL, Shin LM, Lasko NB (2009). Neurobiological basis of failure to recall extinction memory in posttraumatic stress disorder. Biol. Psychiatry.

[12] Kalisch R, Korenfeld E, Stephan KE, Weiskopf N, Seymour B, Dolan RJ (2006). Context-dependent human extinction memory is mediated by a ventromedial prefrontal and hippocampal network. J. Neurosci..

[13] Phelps EA, Delgado MR, Nearing KI, LeDoux JE (2004). Extinction learning in humans: role of the amygdala and vmPFC. Neuron.

[14] Graham BM, Milad MR (2011). The study of fear extinction: implications for anxiety disorders. Am J Psych.

[15] Etkin A, Wager TD (2007). Functional neuroimaging of anxiety: a meta-analysis of emotional processing in PTSD, social anxiety disorder, and specific phobia. Am J Psych.

[16] Milad MR, Orr SP, Lasko NB, Chang Y, Rauch SL, Pitman RK (2008). Presence and acquired origin of reduced recall for fear extinction in PTSD: results of a twin study. J. Psychiatr. Res..

[17] Blechert J, Michael T, Vriends N, Margraf J, Wilhelm FH (2007). Fear conditioning in posttraumatic stress disorder: evidence for delayed extinction of autonomic, experiential, and behavioural responses. Behav. Res. Ther..

[18] Michael T, Blechert J, Vriends N, Margraf J, Wilhelm FH (2007). Fear conditioning in panic disorder: enhanced resistance to extinction. J. Abnorm. Psychol..

[19] Barrett J, Armony J (2009). Influence of trait anxiety on brain activity during the acquisition and extinction of aversive conditioning. Psychol. Med..

[20] Sehlmeyer C, Dannlowski U, Schöning S, Kugel H, Pyka M, Pfleiderer B (2011). Neural correlates of trait anxiety in fear extinction. Psychol. Med..

[21] Soliman F, Glatt CE, Bath KG, Levita L, Jones RM, Pattwell SS (2010). A genetic variant BDNF polymorphism alters extinction learning in both mouse and human. Science.

[22] Chen Z-Y, Jing D, Bath KG, Ieraci A, Khan T, Siao C-J (2006). Genetic variant BDNF (Val66Met) polymorphism alters anxiety-related behavior. Science.

[23] Yu H, Wang Y, Pattwell S, Jing D, Liu T, Zhang Y (2009). Variant BDNF Val66Met polymorphism affects extinction of conditioned aversive memory. J Neuro.

[24] Felmingham KL, Dobson-Stone C, Schofield PR, Quirk GJ, Bryant RA (2013). The brain-derived neurotrophic factor Val66Met polymorphism predicts response to exposure therapy in posttraumatic stress disorder. Biol. Psychiatry.

[25] Zhang L, Benedek D, Fullerton C, Forsten R, Naifeh J, Li X et al. PTSD risk is associated with BDNF Val66Met and BDNF overexpression. Molecular psychiatry. 2013. 10.1038/mp.2012.180 23319005

[26] Dunsmoor JE, Åhs F, LaBar KS. Neurocognitive mechanisms of fear conditioning and vulnerability to anxiety. Front Hum Neurosci. 2011;5. 10.3389/fnhum.2011.00035 PMC3075912 21519378

[27] Indovina I, Robbins TW, Núñez-Elizalde AO, Dunn BD, Bishop SJ (2011). Fear-conditioning mechanisms associated with trait vulnerability to anxiety in humans. Neuron.

[28] Torrents-Rodas D, Fullana MA, Bonillo A, Caseras X, Andión O, Torrubia R (2013). No effect of trait anxiety on differential fear conditioning or fear generalization. Biol Psychol.

[29] Lissek S, Powers AS, McClure EB, Phelps EA, Woldehawariat G, Grillon C (2005). Classical fear conditioning in the anxiety disorders: a meta-analysis. Behav Res Ther.

[30] Dunsmoor JE, Campese VD, Ceceli AO, LeDoux JE, Phelps EA. Novelty-facilitated extinction: providing a novel outcome in place of an expected threat diminishes recovery of defensive responses. Biological Psychiatry. In press. 10.1016/j.biopsych.2014.12.008 PMC4469636 25636175

[31] McEvoy PM, Mahoney AE (2012). To be sure, to be sure: intolerance of uncertainty mediates symptoms of various anxiety disorders and depression. Behav Ther.

[32] Whalen PJ (2007). The uncertainty of it all. Trends Cogn. Sci..

[33] Grupe DW, Nitschke JB (2013). Uncertainty and anticipation in anxiety: an integrated neurobiological and psychological perspective. Nat Rev Neurosci.

[34] van der Heiden C, Muris P, van der Molen HT (2012). Randomized controlled trial on the effectiveness of metacognitive therapy and intolerance-of-uncertainty therapy for generalized anxiety disorder. Behav. Res. Ther..

[35] Dugas MJ, Robichaud M. Cognitive-behavioral treatment for generalized anxiety disorder: From science to practice. Taylor & Francis; 2007

[36] Schiller D, Kanen JW, LeDoux JE, Monfils M-H, Phelps EA (2013). Extinction during reconsolidation of threat memory diminishes prefrontal cortex involvement. Proc. Natl. Acad. Sci..

[37] Delgado MR, Nearing KI, LeDoux JE, Phelps EA (2008). Neural circuitry underlying the regulation of conditioned fear and its relation to extinction. Neuron.

[38] Neumann DL, Waters AM (2006). The use of an unpleasant sound as an unconditional stimulus in a human aversive Pavlovian conditioning procedure. Biol Psychol.

[39] Spielberger CD, Gorsuch RL, Lushene R, Vagg P, Jacobs G. Consulting Psychologists Press, Inc. 2». Palo Alto (CA). 1983.

[40] Meyer TJ, Miller ML, Metzger RL, Borkovec TD (1990). Development and validation of the Penn State worry questionnaire. Behav Res Ther.

[41] Bradley MM, Lang PJ (2007). The international affective digitized sounds (2nd edition; IADS-2): affective ratings of sounds and instruction manual. Technical report B-3.

[42] Watson D, Clark LA, Tellegen A (1988). Development and validation of brief measures of positive and negative affect: the PANAS scales. J. Pers. Soc. Psychol..

[43] Buhr K, Dugas MJ (2002). The intolerance of uncertainty scale: psychometric properties of the English version. Behav Res Ther.

[44] Patton JH, Stanford MS (1995). Factor structure of the Barratt impulsiveness scale. J. Clin. Psychol..

[45] Smith SM (2002). Fast robust automated brain extraction. Hum. Brain Mapp..

[46] Jenkinson M, Bannister P, Brady M, Smith S (2002). Improved optimization for the robust and accurate linear registration and motion correction of brain images. Neuroimage.

[47] Desikan RS, Ségonne F, Fischl B, Quinn BT, Dickerson BC, Blacker D (2006). An automated labeling system for subdividing the human cerebral cortex on MRI scans into gyral based regions of interest. Neuroimage.

[48] Johnstone T, van Reekum CM, Urry HL, Kalin NH, Davidson RJ (2007). Failure to regulate: counterproductive recruitment of top-down prefrontal-subcortical circuitry in major depression. J. Neurol. Sci..

